# Simultaneous use of a heat and moisture exchanger and a heated humidifier causes critical airway occlusion in less than 24 hours

**DOI:** 10.1186/cc14290

**Published:** 2015-03-16

**Authors:** M Mariyaselvam, A Doyle, G Wijewardena, N English, P Young

**Affiliations:** 1 Queen Elizabeth Hospital, King's Lynn, UK

## Introduction

We hypothesised that the simultaneous use of a heat and moisture exchanger (HME) and a heated humidifier (HH) would increase the incidence of airway occlusion over a 24-hour period in comparison with each device in isolation. This bench study compares the incidence of airway occlusion when using (group 1) no airway humidification, (group 2) a HME alone, (group 3) a HH alone and (group 4) both a HME and a HH in combination. Tracheal intubation requires the use of artificial humidification systems. HMEs are less efficient but convenient especially for a short period of intubation and HHs are commonly more expensive. Both devices are often used in close proximity on the ICU depending on the particular clinical scenario and/ or clinical practitioner. Following a critical incident of HME obstruction due to waterlogging on our ICU we realised that HH and HME may be used together inadvertently. This airway obstruction was only resolved by the removal of the HME from the patient's breathing circuit.

## Methods

A lung simulator underwent pressure-controlled ventilation (Pinsp = 25 cmH_2_O; PEEP = 5 cmH_2_O; Vt = 500 ml) for 24 hours for seven test periods for each group (*n *= 24). A HME (Filta-Therm; Intersurgical, Berkshire, UK) was placed between the breathing circuit and catheter mount or the HH (MR850; Fisher & Paykel, Auckland, New Zealand) was used, or both in combination. Circuit manipulation was performed 4-hourly to simulate patient movement. Hourly Vt was recorded to determine airway occlusion. Critical airway occlusion (defined as a drop on the TV to <50 ml) was assessed using a Fisher's exact test.

## Results

In all seven of the breathing circuits in group 4 (both a HME and a HH in combination), critical airway occlusion occurred suddenly between 19 and23 hours. No episodes occurred in the other three groups (*P *< 0.0001).

## Conclusion

The combination of the use of HME and HH within a single ICU risks inadvertent dual use in a single patient and if uncorrected this is likely to result in a ventilator circuit obstruction. Medical errors can be mitigated by consideration of human factors and system engineering to improve patient safety. A focus on clinical awareness and training may lead to improvements; however, the numbers, experience and turnover of critical care staffing would indicate that a systems approach is appropriate and either HME or HH should be used exclusively in an ICU.

